# Similar rates of fat oxidation during graded submaximal exercise in women of different body composition

**DOI:** 10.1371/journal.pone.0242551

**Published:** 2020-11-18

**Authors:** Hugo A. Kerhervé, Leonie M. Harvey, Alexander N. Eagles, Chris McLellan, Dale Lovell

**Affiliations:** 1 School of Sport Science, University of the Sunshine Coast, Sippy Downs, Australia; 2 Univ Rennes, Rennes, France; 3 School of Health and Wellbeing, University of Southern Queensland, Ipswich, QLD, Australia; Universita degli Studi di Roma 'Foro Italico', ITALY

## Abstract

**Background:**

Moderate intensity exercise ranging 40–60% of maximum oxygen uptake is advised to promote energy expenditure and fat oxidation in overweight and obese people. Although fat oxidation has been shown to be highly variable among individual, there is still a relative uncertainty regarding exercise prescription for women specifically. This article aimed to determine whether indicators of body composition can be used to narrow the exercise intensity range for exercise prescription in women.

**Methods:**

A total of 35 healthy women (age 30.8±9.5 yr) classified according to their BMI in normal weight (NOR; ≤24.9 kg·m^2^), overweight (OVW; 25–29.9 kg·m^2^) and obese groups (OBE; ≥30 kg·m^2^) completed a submaximal graded test (intensities eliciting ~30%, 40%, 50% and 60% of maximum oxygen uptake). Blood lactate, perceived exertion and absolute and relative substrate oxidation for fat (OX_FAT_) and carbohydrates (OX_CHO_) were measured at each stage.

**Results:**

Perceived exertion and blood lactate increased as a function of exercise but did not differ across groups. There were no significant changes in absolute and relative OX_FAT_ across groups, or as a function of exercise intensity. Peak OX_FAT_ occurred at the 40%, 50% and 40% stages for NOR, OVW and OBE groups, respectively, with no significant differences across groups.

**Conclusion:**

We measured no differences, but considerable inter-individual variation, in fat oxidation in women of different body composition. This result is in agreement with previous research based on exercise performed at constant rate and in independent participant groups. Our findings do not support the fat oxidation hypothesis, and further emphasise the perspective that exercise prescription should be individualised and likely be based on considerations other than substrate oxidation.

## Introduction

Overweight and obesity is a metabolic condition currently affecting approximately 39% of the adult population worldwide, and between 60% and 65% in North America, Oceania, and most of Western Europe [1]. Overweight and obesity are associated with a substantial economic burden to society [2] from the increased risk of developing a number of chronic diseases, including diabetes, heart disease, hypertension, stroke [3], and an increased risk of mortality [4, 5], especially in the most severe forms of obesity [6]. Overweight and obesity are associated with insulin resistance [7–9] and increased intramuscular triglycerides [8]. A reduced ability to utilise free fatty acids (FFA) as a substrate at rest and during exercise [7, 9] has been cited as a contributing factor to further the progression of obesity [10, 11], although this fat oxidation hypothesis (the inability to utilise fat driving weight gain upward) is criticised [12].

Regular physical activity is advised by the American College of Sports Medicine (ACSM) to promote energy expenditure and contribute to optimal weight loss management in overweight and obese people, with current guidelines recommending up to 250–300 min of moderate-intensity aerobic exercise (40–60% of relative maximum oxygen uptake [${\dot{V}O}_{2MAX}$]) per week [13]. The same relatively broad intensity zone has also been shown to elicit the highest rates of fat oxidation, or peak fat oxidation (PFO), in sedentary and obese individuals [11, 14, 15], and moderate intensity exercise training has been shown to improve fat oxidation and lipolysis [16–19] in obese men [20] and women [21, 22].

Improving the definition of optimal exercise intensity for exercise prescription in overweight and obese people remains a challenge for practitioners. Although increasing total energy expenditure using a wide range of intensities currently is the main known factor informing exercise prescription, the effect of exercise intensity continues to be poorly understood. For instance, while increasing metabolic flexibility could be targeted as a benefit of exercise training, fat oxidation rates vary considerably among individuals and are not well predicted by fat mass [23] as they are influenced by pubertal status [24], training status [25], diet [10, 26], exercise intensity [10, 21, 27] and the mode of exercise that is performed [10, 28]. Furthermore, there is currently little consensus regarding the optimal training intensity specifically in women, despite the specific challenges pregnancy, menopause and contraception impose on cardiovascular health [29]. Despite normal heart rate responses during graded exercise performed at 30–60% of ${\dot{V}O}_{2MAX}$, significantly different blood pressure responses have been reported in obese compared to normal and overweight women [30]. Fat oxidation of independent groups of obese/overweight women has been reported to be lower [31], similar [32–35] or even greater [36] than lean women during constant work rate cycling at discrete exercise intensities from 50% to 65% of ${\dot{V}O}_{2MAX}$. Similarly, substrate oxidation of obese and normal weight women did not differ significantly during constant work rates treadmill exercise at 50%, 70% or 75% of ${\dot{V}O}_{2MAX}$ [37, 38].

Still, there is currently a paucity of research measuring the dynamics of substrate oxidation at various submaximal exercise intensities, in subgroups of women matched for age and predictors of cardiovascular health but differing in body composition. Therefore, this study aimed to determine the dynamics of substrate utilisation as a function of body composition using a submaximal graded treadmill exercise with intensity ranging 30–60% of ${\dot{V}O}_{2MAX}$. From the available literature, it can be hypothesised that substrate oxidation would be characterised by high inter-individual variability, and that fat oxidation would be maximal at intensities 40–60%, with no differences between women of different body composition. Increasing our understanding of the responses to exercise intensity could produce instrumental information destined to clinicians, practitioners and those working in the health and fitness industry.

## Materials & methods

### Participants

Thirty-five healthy female participants were recruited for this study in the local community through the use of advertisements and e-mails (Table 1). Prior to inclusion, participants were informed of the procedures and risks of the study, screened for exclusion criteria (peri- or postmenopausal, currently smoking, pregnant or breast feeding, taking any prescribed medications which may affect heart rate or fat oxidation and excluding the oral contraceptive pill, diabetic, suffering from asthma) and provided written consent. Ethical clearance for this project was granted by the University of the Sunshine Coast ethics committee on human research (project S/11/317).

**Table 1:** Participants’ characteristics. Resting measures of anthropometry and spirometry, and maximum cardiorespiratory and blood lactate measures during the incremental test to exhaustion, of participants classified according to BMI groups.

	Normal	Overweight	Obese	ANOVA	
	<24.9 kg·m^2^ (n = 15)	25–29.9 kg·m^2^ (n = 13)	>30 kg·m^2^ (n = 7)	p	η^2^_p_
**Resting measures**					
Age (yr)	28.3 ± 10.1	33.4 ± 8.9	31.6 ± 8.8	0.362	0.062
Height (m)	1.65 ± 0.05	1.65 ± 0.05	1.68 ± 0.06	0.484	0.044
Weight (kg)	60.9 ± 7.8	**72.3 ± 3.5** ^**A**^ ^**C**^	**104.4 ± 18.4** ^**B**^	**<0.001**	**0.751**
W:H ratio	0.74 ± 0.04	0.77 ± 0.05	0.78 ± 0.04	0.065	0.166
Trunk fat (kg)	5.0 ± 2.5	**8.1 ± 2.2** ^**A**^ ^**C**^	**18.7 ± 5.2** ^**B**^	**<0.001**	**0.760**
Body fat (kg)	17.6 ± 5.7	**25.6 ± 4.0** ^**A**^ ^**C**^	**51.6 ± 10.2** ^**B**^	**<0.001**	**0.798**
Body fat (%)	30.0 ± 7.0	**36.8 ± 5.3** ^**A**^ ^**C**^	**51.2 ± 2.5** ^**B**^	**<0.001**	**0.660**
LBM (kg)	40.2 ± 4.3	43.9 ± 4.2	**49.1 ± 8.5** ^**A**^	**0.020**	**0.230**
Glucose (mmol·L^-1^)	5.19 ± 0.49	5.32 ± 0.44	5.50 ± 0.41	0.348	0.064
Cholesterol (mmol·L^-1^)	4.33 ± 0.72	4.38 ± 0.55	4.44 ± 1.0	0.946	0.003
FVC (L)	3.79 ± 0.39	3.81 ± 0.47	3.76 ± 0.70	0.989	0.001
FEV1 (%)	84.3 ± 6.3	83.4 ± 5.9	84.1 ± 2.1	0.973	0.002
**Incremental test**					
HR (bpm)	188 ± 9	185 ± 7	187 ± 7	0.451	0.049
[La] (mmol·L^-1^)	11.0 ± 1.7	9.5 ± 2.1	**8.4 ± 2.8**^**A**^	**0.026**	**0.205**
VO_2MAX_ (L·min^-1^)	2.34 ± 0.43	2.45 ± 0.49	2.46 ± 0.46	0.791	0.015
VO_2MAX_ (ml·kg·min^-1^)	38.2 ± 7.2	**33.3 ± 5.9** ^**D**^	**23.8 ± 3.7** ^**B**^	**<0.001**	**0.451**
VE_MAX_ (L·min^-1^)	91.9 ± 13.9	93.3 ± 14.5	86.9 ± 13.1	0.613	0.030
RER	1.17 ± 0.06	1.16 ± 0.05	1.13 ± 0.08	0.397	0.056

Data are mean ± SD

^A^ p < 0.05 from BMI <24.9 kg·m^2^

^B^ p < 0.001 from BMI <24.9 kg·m^2^

^C^ p < 0.001 from BMI >30 kg·m^2^

^D^ p < 0.05 from BMI >30 kg·m^2^

Abbreviations used: W:H (waist-to-hip); LBM (lean body mass); FVC (forced vital capacity); FEV1 (forced expiratory volume in 1 s); HR (heart rate), [La] (blood lactate concentration), VO_2MAX_ (maximum oxygen uptake), VE_MAX_ (maximum minute ventilation), RER (respiratory exchange ratio).

Participants reported to the Sunshine Coast University’s exercise physiology laboratory initially for additional screening of medical history (Medical Health Questionnaires), resting measures of pulmonary function using spirometry with forced vital capacity (FVC) and forced expiratory volume (FEV1) manoeuvres, and for assessment of anthropometric characteristics of participants (height, weight, Body Mass Index [BMI; body weight divided by height squared], hip and waist circumferences, waist-to-hip ratio [W:H]), body composition (total and regional body fat, lean body mass [LBM], percentage body fat, bone mineral density and bone mineral content using dual-energy x-ray absorptiometry [DEXA; Lunar Prodigy Advance, GE Healthcare, Buckinghamshire, UK]).

### Overview

This research project consisted of two testing sessions, with at least three days between sessions to minimise the effects of fatigue effects between tests. For both sessions, participants reported to the laboratory after a 10–12 hour overnight fast and no later than 10 am. Blood glucose (Accu-Chek Advantage, Roche Diagnostics, Indianapolis, IN, USA) and cholesterol (Cholestech LDX, Hayward, CA, USA), both measured in millimoles per litre (mmol·L^-1^), were collected using a small sample of capillary blood obtained at the fingertip while the participant was in a fasted state to ensure results were not affected by food ingestion. Exercise was performed using a treadmill (TMX425C, Trackmaster, Full Vision Inc., Newton, KS).

At the first session, participants performed an incremental exercise test to volitional exhaustion to determine workloads associated with ~30%, 40%, 50% and 60% of ${\dot{V}O}_{2MAX}$. After a two minute warm-up walking at 4 km·h^-1^ or 6 km·h^-1^ (depending on the participant’s exercise history and physical characteristics), speed was increased every minute until the participant signalled they had reached a comfortable speed, whereupon the grade of the treadmill was increased by 1–2% every minute until exercise termination. At the second session, participants performed a graded submaximal test with five, 3-min stages (4.5 km·h^-1^ baseline, and treadmill speed×gradient workloads eliciting ~30%, 40%, 50% and 60% of ${\dot{V}O}_{2MAX}$) interspersed with a short rest between stages to allow for the measure of blood lactate. The results of the initial baseline exercise stage are considered as a warmup and are not presented in the results. Although three minute stages may slightly overestimate fat oxidation [39], they have been used in previous research [23, 28] and permit minimising the carryover effects of fatigue.

### Measurements

Ratings of perceived exertion (RPE) were determined at each stage of the graded submaximal test using the 6–20 linear Borg scale. Heart rate (HR) was measured during the incremental test and submaximal graded test at 0.2 Hz using a chest strap (Polar Electro, Kempele, Finland). Blood lactate concentration ([La]) was measured at the fingertip two minutes after exercise termination (session 1) and after each stage (session 2) using a portable analyser (Arkray, Lactate Pro, Kyoto, Japan).

For both tests, the rate of oxygen utilization ($\dot{V}O_{2}$), carbon dioxide production ($\dot{V}{CO}_{2}$), minute ventilation (${\dot{V}}_{E}$), and the respiratory exchange ratio (RER = $\dot{V}O_{2}/\dot{V}{CO}_{2}$) were measured using a two-way, non-rebreathing valve (series 2700, Hans-Rudolph, Kansas City, USA) and an automated open-circuit spirometry metabolic analysis system (True One 2400, Parvo Medics, Sandy UT) with data averaged over 15-s periods. The highest value of $\dot{V}O_{2}$ was used as ${\dot{V}O}_{2MAX}$ provided two or more of the following criteria were met: 1) <2.1 ml·kg^-1^·min^-1^ increase in $\dot{V}O_{2}$ with increasing workload, 2) [La] ≥8 mmol·l^-1^, 3) maximum HR within 10 bpm of their age-predicted maximum HR (based on 220-age), and 4) RER >1.10. All equipments were initially calibrated following standard procedures.

For each intensity of the graded submaximal exercise, absolute (g·min^-1^) and relative (i.e. scaled for LBM: mg·kg LBM^-1^·min^-1^) rates of fat (OX_FAT_) and carbohydrate oxidation (OX_CHO_) were calculated during the last 60-s of each stage using stoichiometric equations [40], based on the assumption that the excretion of urinary nitrogen was negligible. Peak OX_FAT_ is abbreviated as PFO in the following analysis as is customary in the specific literature. Crossover points [41] for each groups were estimated after calculating absolute (kcal·min^-1^) and relative (%) contributions of fat (EE_FAT_) and carbohydrate oxidation (EE_CHO_) to total energy expenditure (EE) using Atwater factors for fat (9 kcal·g^-1^) and carbohydrate (4 kcal·g^-1^).

### Statistical analyses

Participants were classified into three groups based on BMI (normal weight (NOR) ≤24.9 kg·m^2^; overweight (OVW) 25–29.9 kg·m^2^; obese (OBE) ≥30 kg·m^2^). For comparison purposes, participants were also grouped into tertiles of percentage of body fat. Contingency tables showed strong association (p<0.001) between the two approaches and BMI groups were used for analyses.

Statistical tests were performed using the open-access statistical package jamovi [42], with data expressed as mean ± standard deviation (SD), and the level of significance set at p<0.05. An a-priori sample-size analysis revealed the study design could reliably detect significant differences between independent groups of n = 7 with moderate to large effect sizes (d>0.5) with a probability greater than 0.8, assuming a two-sided criterion allowing for a maximum Type I error rate of α = 0.05. Differences between groups (NOR, OVW, OBE) in resting characteristics (anthropometry, body composition, blood glucose and cholesterol, spirometry), in cardiorespiratory and [La] measures during the incremental test, in absolute and relative PFO and crossover points during the submaximal graded test, were determined using one-way ANOVAs with Bonferroni post-hoc tests. The effect of exercise intensity (30%, 40%, 50% and 60%), BMI group (NOR, OVW, OBE) and their interaction (intensity×group) on RPE, [La], absolute and relative OX_FAT_ were assessed using 2-way, repeated measures ANOVAs, with Bonferroni post-hoc tests. Pearson’s r was used to assess relationships between PFO (absolute and relative), age, body fat, LBM and absolute $\dot{V}O_{2MAX}$. To identify the main predictors of substrate utilisation, a multiple linear regression was then performed between absolute OX_FAT_ and covariates age, body fat, LBM, and absolute $\dot{V}O_{2MAX}$. For ANOVAs, asssumptions of equality of variances were initially checked using Levene’s test, and assumptions of sphericity were checked using Mauchly’s W with Greenhouse-Geisser corrections if significant. For correlations, assumptions of normality were initially checked. We reported effect sizes for ANOVAs using partial eta-squared (η^2^_p_) interpreted according to Cohen’s scale (small effect: 0.01<η^2^_p_<0.06, medium effect: 0.06<η^2^_p_<0.14, and large effect: η^2^_p_>0.14).

## Results

All participants completed the study procedures. There were no significant differences between participant groups in age, height, and pulmonary function (Table 1). Participants in OVW group were characterised by higher weight, body and trunk fat compared to NOR (Table 1). Participants in OBE group were characterised by higher weight, body fat and trunk fat compared to both OVW and NOR, and higher LBM compared to NOR only (Table 1). During the incremental test, participants in OBE group had significantly lower maximum [La] concentrations compared to NOR, and significantly lower relative $\dot{V}O_{2MAX}$ compared to NOR and OVW (Table 1). There were no other significant differences in maximum absolute $\dot{V}O_{2}$, VE or HR across participant groups (Table 1).

Speed, gradient, RPE, blood [La] and absolute and relative OX_FAT_ measured during each stage of the submaximal test are provided in Table 2. There was a significant and large main effect of exercise on RPE (p<0.001; η^2^_p_ = 0.803), with no effect of group (p = 0.799; η^2^_p_ = 0.014) or interaction effects (p = 0.728; η^2^_p_ = 0.036). There was also a significant and large main effect of exercise on [La] (p<0.001; η^2^_p_ = 0.490), with no group (p = 0.560; η^2^_p_ = 0.036) or interaction effects (p = 0.206; η^2^_p_ = 0.083). For absolute PFO, there were no significant effects or exercise (p = 0.522; η^2^_p_ = 0.026), group (p = 0.124; η^2^_p_ = 0.139) or interaction (p = 0.861; η^2^_p_ = 0.029). For relative OX_FAT_, there were also no significant effects of exercise (p = 0.765; η^2^p = 0.015), group (p = 0.162; η^2^_p_ = 0.131) or interaction (p = 0.998; η^2^_p_ = 0.006). PFO occurred at 50%, 40% and 50% for NOR, OVW and OBE groups, respectively, with no statistically significant effect of group for absolute (p = 0.113; η^2^_p_ = 0.127) or relative measures (p = 0.283; η^2^_p_ = 0.081).

**Table 2:** Physiological responses during the submaximal graded test. Speed, gradient, ratings of perceived exertion, blood lactate concentration and absolute and relative substrate oxidation of participants classified according to BMI groups.

	Normal	Overweight	Obese
	<24.9 kg·m^2^ (n = 15)	25–29.9 kg·m^2^ (n = 13)	>30 kg·m^2^ (n = 7)
**Speed (km·hr**^**-1**^**)**			
30%VO_2MAX_	4.6 ± 1.0	4.0 ± 0.8	2.9 ± 0.5
40%VO_2MAX_	5.6 ± 0.9	5.1 ± 0.8	3.7 ± 1.0
50%VO_2MAX_	6.6 ± 1.0	6.1 ± 0.9	4.9 ± 0.7
60%VO_2MAX_	7.5 ± 1.2	6.9 ± 0.7	5.7 ± 0.6
**Gradient (%)**			
30%VO_2MAX_	0.5 ± 0	0.5 ± 0	0.5 ± 0
40%VO_2MAX_	0.5 ± 0	0.5 ± 0	0.5 ± 0
50%VO_2MAX_	0.7 ± 0.5	0.5 ± 0	0.5 ± 0
60%VO_2MAX_	1.1 ± 1.4	1.0 ± 0.9	0.5 ± 0
**RPE**			
30%VO_2MAX_	7.2 ± 1.4	7.3 ± 1.8	6.1 ± 0.4
40%VO_2MAX_	9.0 ± 2.2	9.3 ± 2.0	8.3 ± 1.9
50%VO_2MAX_	11.0 ± 2.0	11.1 ± 2.1	10.9 ± 2.4
60%VO_2MAX_	12.7 ± 2.2	12.9 ± 2.4	13.5 ± 1.4
**[La] (mmol·L**^**-1**^**)**			
30%VO_2MAX_	1.3 ± 0.3	1.3 ± 0.4	1.5 ± 0.7
40%VO_2MAX_	1.4 ± 0.4	1.4 ± 0.4	1.5 ± 0.7
50%VO_2MAX_	2.0 ± 0.6	1.6 ± 0.6	1.7 ± 1.0
60%VO_2MAX_	2.9 ± 0.9	2.5 ± 1.0	2.2 ± 1.3
**OX**_**FAT**_ **(g·min**^**-1**^**)**			
30%VO_2MAX_	0.301 ± 0.093	0.262 ± 0.093	0.322 ± 0.103
40%VO_2MAX_	0.312 ± 0.103	0.272 ± 0.106	0.359 ± 0.072
50%VO_2MAX_	0.319 ± 0.087	0.254 ± 0.133	0.387 ± 0.032
60%VO_2MAX_	0.298 ± 0.145	0.258 ± 0.206	0.379 ± 0.112
**OX**_**FAT**_ **(mg·kg LBM**^**-1**^**·min**^**-1**^**)**			
30%VO_2MAX_	7.41 ± 2.08	5.94 ± 2.00	6.69 ± 1.86
40%VO_2MAX_	7.71 ± 2.49	6.20 ± 2.17	7.28 ± 1.67
50%VO_2MAX_	7.86 ± 1.91	5.60 ± 2.90	8.25 ± 1.73
60%VO_2MAX_	6.84 ± 3.75	5.90 ± 4.42	8.05 ± 2.11

Data is displayed as mean ± SD

Abbreviations used: RPE (ratings of perceived exertion), [La] (blood lactate concentration), OX_FAT_ (fat oxidation), EE_FAT_ (relative contribution of fat oxidation to total energy expenditure)

**Fig 1:**
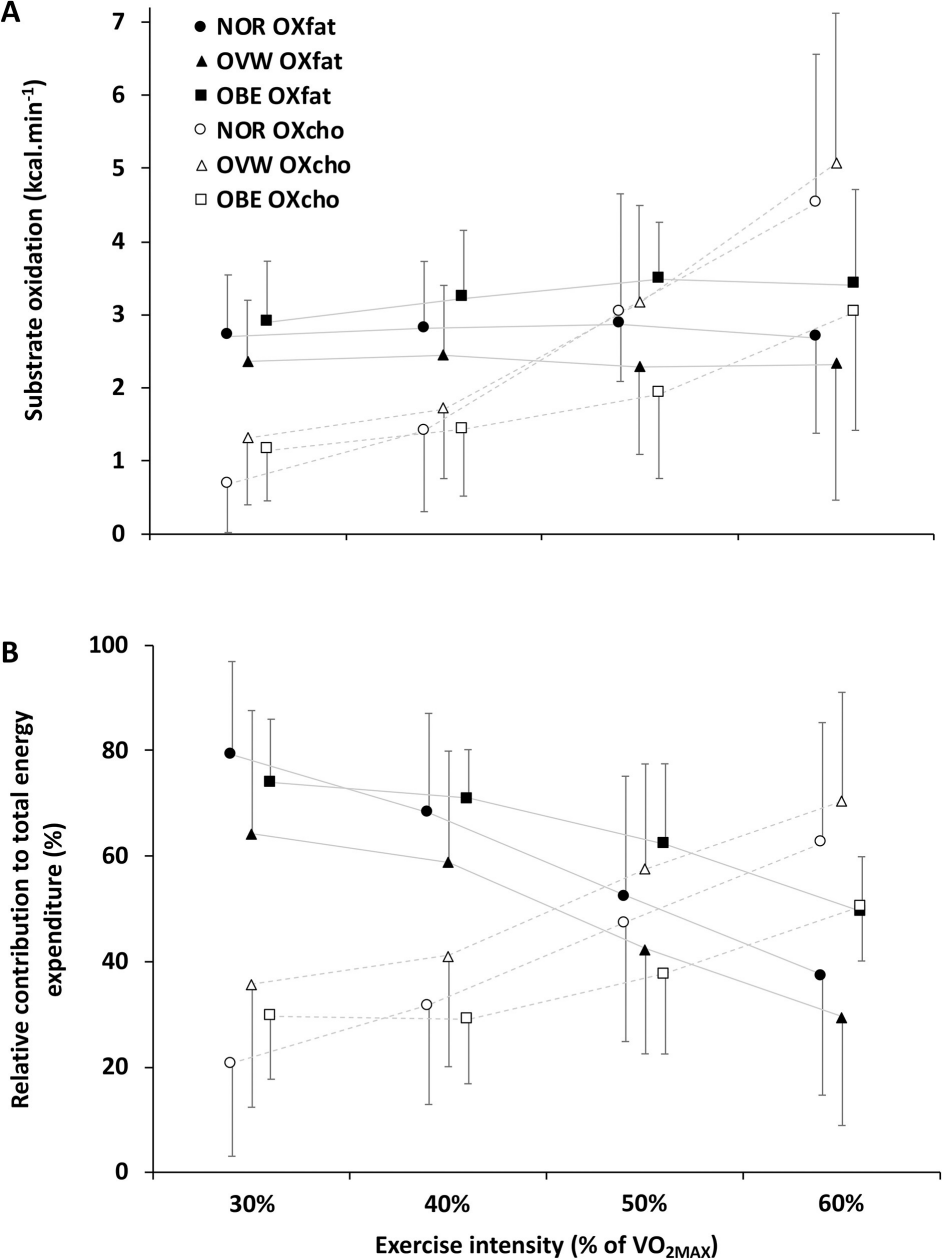
Substrate oxidation and energy expenditure. Absolute substrate oxidation (panel A) and relative contributions to total energy expenditure (panel B) for fat (OX_FAT_) and carbohydrate oxidation (OX_CHO_) during the submaximal graded test of participants classified according to BMI groups (NOR <24.9 kg·m^2^; OVW = 25–29.9 kg·m^2^; OBE >30 kg·m^2^).

Absolute (kcal·min^-1^) and relative (%) EEFAT and EECHO are presented (Fig 1). Crossover point was estimated to occur at 50.6±6.8%, 48.3±8.2% and 55.1±8.2% of ${\dot{V}O}_{2MAX}$ for NOR, OVW and OBE groups, respectively, with no statistically significant differences across groups (p = 0.226; η^2^_p_ = 0.108).

For absolute PFO, correlations were statistically significant with absolute $\dot{V}O_{2MAX}$ (r = 0.490; p<0.001), and LBM (r = 0.374; p = 0.032), but not with age (r = -0.162; p = 0.353) and fat mass (r = 0.190; p = 0.290). For relative PFO, correlations were not statistically significant with absolute $\dot{V}O_{2MAX}$ (r = 0.219; p = 0.221), age (r = -0.172; p = 0.339), LBM (r = -0.047; p = 0.797) and fat mass (r = -0.049; p = 0.785). Significant relationships were also observed between age and absolute $\dot{V}O_{2MAX}$ (r = -0.409; p = 0.015) and body fat (r = -0.791; p<0.001). The multiple linear regression for absolute peak OX_FAT_ with the covariates absolute $\dot{V}O_{2MAX}$ (standardised estimate [SE] = 0.588; p = 0.039), age (SE = 0.006; p = 0.997), body fat (SE = -0.158; p = 0.615) and LBM (SE = 0.291; p = 0.228) was not significant (p = 0.905; r = 0.529, R^2^ = 0.280).

## Discussion

The aim of this study was to compare substrate utilization during submaximal treadmill exercise in women classified as normal, overweight and obese BMI. The main finding of this project was that there were no differences in fat oxidation in 35 women of normal, overweight and obese BMI, aged 18–50 years, with no underlying health conditions.

In agreement with previous literature [23], we observed very large inter-individual variation in substrate oxidation rates (both absolute and relative to lean body mass) independent of body composition in a graded exercise ranging 30–60% of maximum aerobic capacity. While no previous study has specifically compared PFO between normal, overweight and obese women 18–50 years of age, our results are consistent with the absolute [11, 38] and relative fat oxidation rates [23] observed in similar participant groups independently. Similarly, substrate oxidation relative to total energy expenditure was not different across groups of BMI, albeit with important inter-individual variability. Furthermore, while the relative contribution of fat oxidation to total energy expenditure decreased after 50% in all groups, absolute fat oxidation remained unaffected by exercise intensity, including at 60%.

A significant strength of the current study is the assessment of body composition using DEXA to ensure all participants were appropriately grouped into the correct BMI category: while average age and height of each BMI category were not different across groups, weight was significantly greater in OVW compared to NOR, and greater in OBE compared to OVW. In agreement with previous research [11], we also measured higher body fat (absolute and relative) in OBE compared to NOR, and in OVE compared to NOR. We also measured higher trunk fat in OBE compared to NOR, and in OVE compared to NOR, although W:H ratio was not significantly different across groups. Importantly, both body fat and trunk fat are associated with increase the risk people to develop further health problems such as metabolic syndrome and cardiovascular disease [13].

In this study, simple linear regressions indicate that the main determinants of absolute PFO were $\dot{V}O_{2MAX}$ and LBM, although the regression model failed to reach significance. While ${\dot{V}O}_{2MAX}$ was negatively correlated with age and body fat, we did not find age to be a significant predictor of OX_FAT_ during exercise, which could differ from conclusions drawn from OX_FAT_ measured at rest [43]. We also measured no differences between groups in blood glucose and cholesterol at rest, or differences between groups in blood lactate concentration or in ratings of perceived exertion during the graded treadmill exercise, despite differences in relative ${\dot{V}O}_{2MAX}$ and lactate concentration during the incremental test. As such, our results confirm the exercise was truly submaximal, and are consistent with recent literature having determined that aerobic fitness (here represented as $\dot{V}O_{2MAX}$) is a greater contributor to PFO than dietary intake [44].

The findings of the current study are therefore in agreement with an existing body of literature underscoring that marked inter-individual variations in fat oxidation exist between healthy women of different body composition, and that establishing a substrate oxidation profile by increasing the number of exercise intensities tested did no modify the expected outcomes that can be inferred from the literature. Further, low power correlations existed between fat oxidation and typical predictors of cardiorespiratory fitness. Lastly, exercise intensity had no significant bearing on fat oxidation and therefore, exercise prescription should be individualised and likely be based on considerations other than substrate oxidation (at least during submaximal exercise). As such, the popularity of high-intensity interval exercise provides interesting avenues for further research in substrate oxidation as a function of body composition, especially considering the effect of exercise intensity of post-exercise energy consumption and thermogenesis.

It is important to note that our study had four main limitations. The first limitation is that menstrual phase was not accounted for in the present investigation, however, all participants were required to have regular menstruation and not present with any symptoms suggesting menopause. Menstrual phase is a contentious issue regarding substrate utilisation, with conflicting findings being reported throughout the literature, and should be further investigated. The second limitation is that the obese BMI group counted only 7 women in the current study, which could have blunted true population variance. However, inter-individual variations were similar across the three BMI groups accounting for 35 women in total, and we measured high association between groups of BMI and participants grouped by tertiles of body mass. Therefore, and in agreement with previous studies using constant work rates in independent groups, it is unlikely that increasing our participant pool would have affected our main finding. Thirdly, the graded submaximal test used a warmup stage followed by consecutive stages of increasing difficulty as priming exercises to reach steady state as quickly as possible. Although we mitigated the risk of fatigue carryover between stages by including a rest period between each stage (all inferior to 2-min), it is possible that the later stages were affected by exercise-induced fatigue. Additionally, there is evidence 3-min tests may be too short in very sedentary participants to establish a reliable measure of fat oxidation [39]. Nonetheless, the graded test was tolerated by all participants, performed at truly submaximal intensities, and all our results are consistent with previous literature. Still, randomized intensity stages may be a useful addition to future studies using graded tests for similar purposes. Lastly, we did not quantitatively control for habitual diet and physical activity before the submaximal test, which are factors known to affect measures of macronutrient contribution to total energy energy expenditure to some extent [12].

## Conclusions

In agreement with the literature, this study reports that there were no differences in fat oxidation (absolute and relative to lean body mass) during submaximal exercise performed in a fasted state in women differing in body composition, with peak fat oxidation occurring at 40–50% and crossover point estimated at 48–55% of ${\dot{V}O}_{2MAX}$. Therefore, specific exercise intensities are not influenced by body composition and our findings do not support the fat oxidation hypothesis. Appropriate exercise prescription should emphasize individualizing testing responses and likely be based on considerations other than substrate oxidation for efficient weight management.

## Supporting information

S1 Dataset https://doi.org/10.6084/m9.figshare.12783347.v1. (TXT)

S1 Questionnaires https://doi.org/10.6084/m9.figshare.13043009.v1. (TXT)
